# To Determine the Efficacy of the Modified Shock Index in Predicting the Need for Blood Transfusions in Patients With Multiple Injuries

**DOI:** 10.7759/cureus.93558

**Published:** 2025-09-30

**Authors:** Shaurav Ghosh

**Affiliations:** 1 General Surgery, East Point College of Medical Sciences and Research Centre, Bengaluru, IND

**Keywords:** critical hemorrhagic shock, length of hospital stay (los), massive blood transfusion, modified shock index, mortality rate in icu, multiple trauma, trauma and injury severity score (triss)

## Abstract

Introduction: Excessive blood loss remains a major cause of early mortality in trauma patients. Prompt recognition of circulatory failure is crucial for initiating life-saving interventions. However, standard clinical signs, such as heart rate and blood pressure, often remain within normal limits during the initial compensatory phase, thereby masking the early signs of shock. The Shock Index (SI), which is the ratio of heart rate to systolic blood pressure, is widely used to evaluate cardiovascular status; however, it may not accurately reflect tissue perfusion. To improve sensitivity, the Modified Shock Index (MSI) replaces systolic pressure with mean arterial pressure, offering a potentially more accurate measure of hemodynamic compromise.

Aim and objective: The aim of our study was to assess the predictive value of MSI for early blood transfusion requirements in polytrauma patients and to evaluate its association with adverse outcomes, including ICU admission, need for mechanical ventilation, and mortality.

Methods: This prospective observational study evaluated MSI as a predictor of transfusion requirements and clinical outcomes in 63 polytrauma patients. MSI was calculated at admission, and outcomes, including transfusion >1500 mL, ICU admission, mechanical ventilation, and mortality, were recorded.

Results: The mean MSI at admission was significantly higher in patients who required transfusion volumes greater than 1500 mL. An MSI >1.4 predicted massive transfusions with an AUROC (area under the receiver operating characteristic curve) of 0.890, a sensitivity of 82.5%, and a specificity of 95.65%. Multivariate analysis confirmed MSI > 1.3 as an independent predictor of high transfusion needs (AOR 67.20; p < 0.001). Higher MSI also correlated with adverse outcomes.

Conclusion: The MSI may serve as a simple, non-invasive bedside tool with potential value for early risk stratification in trauma patients. However, given the single-center observational design of this study, these findings demonstrate association rather than causation, and further multicenter studies with larger cohorts are needed to validate these findings and support the integration of MSI thresholds into standardized trauma management guidelines.

## Introduction

Significant hemorrhage is a primary contributor to early death in trauma cases, especially before patients reach the hospital. While shock typically occurs after losing around 30-40% of the circulating blood volume, this is not an absolute cutoff. Some individuals may show shock symptoms sooner, highlighting that, although blood loss is a common cause, it is not always the sole predictor of shock. The Advanced Trauma Life Support (ATLS) classification associates Class III hemorrhage (30-40% blood loss) with hypotension and altered mental status, while emphasizing the wide variability in physiological responses among patients [1-3]. For the initial evaluation of circulatory depletion, ATLS delineates four stages of hypovolemic shock, with management ranging from crystalloid resuscitation (Classes I-IV) to blood product transfusion (Classes III-IV). These guidelines are based on the empirically derived "3:1 rule," which proposes that approximately 300 mL of crystalloid is required for every 100 mL of blood lost [3,4].

Current guidelines emphasize minimizing crystalloid use and instead recommend early administration of blood products in balanced ratios (e.g., 1:1:1 for packed red blood cells (PRBCs), plasma, and platelets), in line with damage-control resuscitation principles. The PROPPR trial and subsequent trauma guidelines support this strategy, demonstrating reduced exsanguination-related mortality. Furthermore, permissive hypotension, particularly in penetrating trauma with ongoing hemorrhage, has been shown to improve outcomes by limiting bleeding and preserving coagulation. Excessive crystalloid administration is now recognized to cause adverse effects, including coagulopathy, hypothermia, and increased mortality; therefore, current practice discourages large-volume resuscitation in favor of judicious fluid use with frequent reassessment [4]. Nevertheless, reliance on conventional vital signs alone may fail to detect early circulatory compromise, potentially delaying timely intervention [5,6].

To address this, Allgöwer and Burri introduced the Shock Index (SI), a simple, early indicator of hemodynamic instability in trauma patients [6]. A recent systematic review and meta-analysis by Carsetti et al. confirmed that SI is a reliable predictor of both massive transfusion requirements and mortality in trauma patients, reinforcing its value as an early triage tool [7]. Recognizing the limitations of relying solely on systolic blood pressure, Liu et al. proposed the Modified Shock Index (MSI), which incorporates mean arterial pressure (MAP), and demonstrated its improved predictive accuracy for mortality in emergency settings [8]. MSI has been shown to be a clinically significant predictor of mortality and may perform better than heart rate or blood pressure alone. In contrast, the conventional SI was not found to be significantly correlated with mortality in emergency patients [8].

Gonzalez et al. further highlighted the rapid and fatal progression of hemorrhagic shock, especially in pediatric trauma, underscoring the need for early identification and targeted resuscitation [9]. Building on this evidence, the present study evaluates the predictive accuracy of the MSI in identifying transfusion requirements among polytrauma patients, while recognizing that MSI should serve as an adjunct rather than a standalone tool in predicting massive transfusion.

## Materials and methods

Study design and setting

This prospective study was conducted over a 12-month period (February 2023 to January 2024) at a tertiary trauma center. All adult trauma patients presenting to the emergency department (ED) were screened for eligibility. Institutional Ethics Committee approval was obtained before study initiation.

Inclusion and exclusion criteria

Eligible trauma patients were enrolled consecutively upon ED admission. Unconscious patients were included after informed consent from a legal representative. Exclusion criteria included patients <18 years, death within six hours, incomplete clinical data, isolated head injuries, non-traumatic conditions (e.g., burns, drowning, overdose), and trauma in pregnancy.

Data collection

Estimation of Blood Loss in Trauma

Blood loss in trauma was estimated using a multimodal approach incorporating both clinical and objective parameters. The following methods were employed: (1) Clinical assessment: Hemorrhage severity was classified according to the ATLS framework, using core physiological indicators including heart rate, blood pressure, respiratory rate, mental status, and urine output, which correspond to hemorrhage Classes I-IV. (2) Visual estimation: Gross blood loss was approximated based on visible contamination of patient clothing, floor surfaces, and absorbent materials (e.g., gauze, dressings). This technique, while frequently used in emergency care, is recognized as being imprecise and particularly prone to underestimation in the context of severe hemorrhage. (3) Physiological indices: Calculations of the SI and the MSI were used to provide indirect, quantitative markers of circulatory compromise. (4) Laboratory parameters: Serial measurements of hemoglobin and hematocrit, along with metabolic markers such as base deficit and serum lactate, were monitored as surrogate indicators of ongoing blood loss and systemic hypoperfusion. (5) Intraoperative/procedural quantification: Direct measurements were obtained intraoperatively using suction canister volumes and gravimetric assessment of sponges and surgical materials. (6) Transfusion requirements: In alignment with established trauma literature, the administration of ≥10 units of PRBCs within the first 24 hours was employed as a surrogate marker for massive hemorrhage.

All participating personnel, including nursing staff, residents, attending physicians, and emergency medical responders, received pre-study training in the application of these assessment methods. Despite these measures, the potential for observer bias could not be fully eliminated and is acknowledged as a methodological limitation.

Patients were selected using simple random sampling. Clinical and demographic data, including age, sex, heart rate, blood pressure, and estimated blood loss, were recorded at the time of admission. Vital signs were monitored for six hours, with SI (SI = HR/SBP) and MSI (MSI = HR/MAP; MAP = [(2 × DBP) + SBP]/3) recalculated at each interval. Thresholds were set for HR >120 bpm, SBP <90 mmHg, MAP <100 mmHg, SI <0.6 or ≥1.4, and MSI <0.7 or >1.3.

Patients were classified according to ATLS guidelines and the injury severity using the Injury Severity Score (ISS). SI and MSI were analyzed as predictors of transfusion requirements and adverse outcomes.

Statistical methods

This study employed both descriptive and inferential statistics. Continuous variables are presented as mean ± standard deviation (SD) with minimum and maximum values, while categorical variables are expressed as a number (percentage). Statistical significance was interpreted as follows: p > 0.05, not significant; 0.05 < p ≤ 0.10, suggestive significance; 0.01 < p ≤ 0.05, moderately significant; p ≤ 0.01, strongly significant; and p < 0.001, highly statistically significant. Analyses assumed that the dependent variables were normally distributed, that the samples were randomly selected, and that the observations were independent. An independent two-tailed Student's t-test was used to compare continuous variables between two groups, with Levene's test assessing the equality of variances. This test evaluates whether the observed difference in group means is statistically significant under the null hypothesis of no difference. Categorical variables were analyzed using the chi-square test or Fisher's exact test, as appropriate, to examine associations within qualitative data [10,11].

Fisher's exact test was applied when expected cell counts were below five, where the chi-square test loses accuracy. Yates' correction was used to reduce the risk of Type I error by making the test more conservative [12,13].

Because MSI is mathematically derived from HR and MAP, potential collinearity with its individual components was considered [6,7]. To address this, HR, MAP, and MSI were not entered simultaneously into the same regression model; instead, separate models were constructed to maintain independence. Collinearity was further assessed using the variance inflation factor (VIF) analysis, with all values <2, indicating no significant multicollinearity [13].

A Receiver Operating Characteristic (ROC) curve plots the true positive rate (sensitivity) against the false positive rate (1-specificity) across different thresholds, illustrating diagnostic performance. Each point reflects a specific sensitivity/specificity pair. The Area Under the Curve (AUC) quantifies overall accuracy: AUC 0.9-1.0 is excellent, 0.8-0.9 good, 0.7-0.8 fair, 0.6-0.7 poor, and 0.5-0.6 indicates no discriminative ability. Likelihood Ratios (LRs) assess the diagnostic impact of test results: LR >10 greatly increases disease likelihood, 5-10 is moderate, and 2-5 is small; values near 1 suggest minimal impact. LR < 1 decreases likelihood: 0.5-1 slight, 0.2-0.5 small, 0.1-0.2 moderate, and < 0.1 a strong, often conclusive, decrease. Logistic regression was used to evaluate associations between clinical variables and positivity (Adj OR = 1: no association; >1: positive; <1: negative) [14,15].

The collected data were subsequently analyzed using IBM SPSS Statistics for Windows, Version 22 (Released 2013; IBM Corp., Armonk, New York) and the R statistical environment version 3.2.2, an open-source software. Microsoft Excel and Word (Microsoft Corporation, Redmond, Washington) were utilized to generate tables, graphs, and other data visualizations.

## Results

Table 1 shows that the majority of trauma patients were young adults, with 47.6% aged between 20 and 30 years, and 20.6% between 1 and 40 years. The mean age was 30.03 ± 12.42 years, indicating a predominantly young cohort with a wide age range.

**Table 1:** Age in years frequency distribution of the patients studied A one-sample Student's t-test (t(62) = –3.176, p = 0.0023, two-tailed) was used to compare the mean age (30.03 ± 12.42 years) with a hypothesized value of 35 years, showing a statistically significant difference (p < 0.05). A chi-square goodness-of-fit test (χ² = 33.11, df = 4, p < 0.001) was used to compare age group frequencies against a uniform distribution, demonstrating a highly significant deviation [10,11].

Age in Years	No. of Patients, n=63	Total N=100%	Test Statistic	P-value
<20	9	14.3	-	-
20–30	30	47.6	-	-
31–40	13	20.6	-	-
41–50	6	9.5	-	-
>50	5	7.9	-	-
Mean ± SD	-	30.03 ± 12.42	t(62) = –3.176	0.0023 (significant)
Age distribution (overall)	-	-	χ² = 33.11, df = 4	<0.001 (highly significant)
Total	63	100	-	-

Table 2 reveals a notable gender imbalance, with 85.7% males and 14.3% females.

**Table 2:** Gender frequency distribution of the patients studied Gender distribution differed significantly from an expected equal male-to-female ratio (chi-square goodness-of-fit: χ² = 32.14, df = 1, p < 0.001, highly significant), with males markedly overrepresented [10,11].

Gender	No. of Patients, n=63	Total N=100%	Test Statistic	P-value
Female	9	14.3	-	-
Male	54	85.7	-	-
Total	63	100	χ² = 32.14, df = 1	<0.001 (highly significant)

The hospital length of stay is summarized in Table 3, where most patients had short admissions: 34.9% stayed 1-2 days, and 25.4% stayed 3-5 days. The mean stay duration was 6.28 days, likely influenced by variations in injury severity and clinical progression.

**Table 3:** Length of hospital stay (LOHS in days) frequency distribution of the patients studied The mean LOHS was 6.28 ± 7.40 days, and the chi-square goodness-of-fit test (χ² = 30.05, df = 5, p < 0.001) showed a highly significant deviation from a uniform distribution, with discharges clustering in the early days [10-12].

LOHS (Days)	No. of Patients, n = 63	Total N=100%	Test Statistic	P-value
0	3	4.8	-	-
1–2	22	34.9	-	-
3–5	16	25.4	-	-
6–10	14	22.2	-	-
10–20	4	6.3	-	-
>20	4	6.3	-	-
Total	63	100	χ² = 30.05, df = 5	p < 0.001 (highly significant)
Mean ± SD: 6.28±7.40				

According to Table 4, non-operative management was more common (54.0%) than surgical intervention (41.3%). The overall mortality rate was 4.8%. In our cohort, the majority of patients were managed non-operatively, reflecting the institutional protocol that favors conservative treatment when hemodynamic stability and injury patterns allow.

**Table 4:** Treatment plan frequency distribution of the patients studied The chi-square goodness-of-fit test for the treatment plan yielded χ² = 24.67, df = 2, and p < 0.001, indicating a highly significant deviation from a uniform distribution, with most patients managed non-operatively [10-12]. Tx: Treatment Plan, DECD: Deceased, NOM: Non-Operative Management, OP: Operated

Tx Plan	No. of Patients, n = 63	Total N=100%	Test Statistic	P-value
DECD	3	4.8	-	-
NOM	34	54	-	-
OP	26	41.3	-	-
Total	63	100	χ² = 24.67, df = 2	< 0.001 (highly significant)

Table 5 indicates that nearly 69.8% of patients had an ISS greater than 25, reflecting a high burden of trauma in this cohort.

**Table 5:** Injury severity score frequency distribution of the patients studied The distribution of Injury Severity Scores [16-18] showed a significant deviation from a uniform distribution across categories (chi-square goodness-of-fit test: χ² = 78.57, df = 3, p < 0.001 (highly significant)), with the majority of patients (69.8%) having scores >25, indicating severe injury burden in the cohort [10-12]. ISS: Injury Severity Scores

ISS Category	No. of Patients, n=63	Total N=100%	Test Statistic	P-value
1–8	0	0	-	-
9–15	3	4.8	-	-
16–24	16	25.4	-	-
>25	44	69.8	-	-
Total	63	100	χ² = 58.19, df = 3	p < 0.001 (highly significant)

Consistent with this, Table 6 shows that while many patients experienced mild blood loss (0-750 mL), a significant number had losses exceeding 2000 mL, highlighting considerable variability in the severity of hemorrhage among the patients.

**Table 6:** Blood loss frequency distribution of the patients studied Advanced Trauma Life Support (ATLS) classification of hemorrhagic shock in trauma patients [3,5]. Chi-square goodness-of-fit test: χ² = 1.38, df = 3, p = 0.709, not significant (NS), indicating no statistically significant deviation from an equal distribution across categories [10,11].

Blood Loss, mL	No. of Patients, n=63	Total N=100%	Test Statistic	P-value
0–750 mL	19	30.2	-	-
750–1500 mL	15	23.8	-	-
1500–2000 mL	15	23.8	-	-
>2000 mL	14	22.2	-	-
Total	63	100	χ² = 1.38, df = 3	p = 0.710 (NS)

Table 7 shows vital sign abnormalities common in transfused patients: tachycardia (>120 bpm), systolic BP <140 mmHg, diastolic BP <90 mmHg, and MAP <100 mmHg, indicating early hemodynamic compromise.

**Table 7:** Distribution of hemodynamic parameters in the study population Chi-square goodness-of-fit tests were used to compare observed frequencies for each parameter against an expected equal distribution [10,11].

Variables	No. of Patients, n=63	Total N=100%	Test Statistic	P-value
HR (beats/min)				
<120	21	33.3	χ² = 7.14, df = 1	p = 0.008 (significant)
≥120	42	66.7		
SBP (mmHg)				
<140	44	69.8	χ² = 10.19, df = 1	p = 0.001 (highly significant)
≥140	19	30.2		
DBP (mmHg)				
<90	46	73.00	χ² = 14.27, df = 1	p < 0.001 (highly significant)
≥90	17	27.00		
Mean Arterial Pressure (mmHg)				
<100	46	73.00	χ² = 14.27, df = 1	p < 0.001 (highly significant)
≥100	17	27.00		
Total	63	100.00	-	-

Table 8 demonstrates that the majority of patients fall into higher SI categories (Group IV SI ≥1.4 and MSI >1.3), aligning with the high rates of transfusion (63.5%) and surgical intervention or invasive intervention (53.9%). These trends reflect the severity of trauma and physiological derangement in this patient cohort and justify the need for early aggressive resuscitation and decision-making tools.

**Table 8:** Clinical correlation between shock indices, ISS, and transfusion requirement Chi-square tests for association between clinical outcomes and TR-DGU SI groups [19], MSI, ATLS classification, and patient outcomes. Significant associations were observed for TR-DGU SI groups, ICU admission, and mortality. TR-DGU shock index groups showed a significant deviation from a uniform distribution (χ² = 24.89, df = 3, p < 0.001), with the majority of patients in Group IV (SI ≥ 1.4). ICU admission (χ² = 8.19, df = 1, p = 0.004) and mortality (χ² = 53.38, df = 1, p < 0.001) were also significantly different from expected frequencies, while other variables showed no significant differences, p > 0.05 [10,11].

Variable	Category	No. of Patients, n=63	Total N=100%	Test Statistic	P-value
TR-DGU (Shock Index groups)	Group I, SI <0.6	1	1.6	χ² = 24.89, df = 3	p < 0.001 (highly significant)
	Group II, SI ≥0.6 to <1.0	19	30.2		
	Group III, SI ≥1.0 to <1.4	18	28.6		
	Group IV, SI ≥1.4	25	39.7		
Modified Shock Index (MSI)	<0.7	0	0.00	χ² = 2.68, df = 1	p = 0.102 (NS)
	0.7–1.3	25	39.7		
	>1.3	38	60.3		
ATLS Classification	Class I	16	25.4	χ² = 0.29, df = 3	p = 0.962 (NS)
	Class II	18	28.6		
	Class III	14	22.2		
	Class IV	15	23.8		
Blood transfusion	Yes	40	63.5	χ² = 2.54, df = 1	p = 0.111 (NS)
Surgery/procedure	Yes	34	53.9	χ² = 0.02, df = 1	p = 0.884 (NS)
ICU admission	Yes	44	69.8	χ² = 8.19, df = 1	p = 0.004 (significant)
Sepsis	Yes	22	34.9	χ² = 3.10, df = 1	p = 0.078 (NS)
Mechanical ventilation	Yes	31	49.2	χ² = 0.06, df = 1	p = 0.805 (NS)
Mortality	Yes	3	4.8	χ² = 53.38, df = 1	p < 0.001 (highly significant)

Table 9 compares continuous clinical variables (ISS, SI, MSI) between ICU and non-ICU patients. All variables showed statistically significant differences (p < 0.001), with higher values in ICU patients. This highlights the association of elevated ISS, SI, and MSI with the need for intensive care, supporting their role as early predictors of critical illness in trauma. Patients who required blood transfusion had a significantly higher mean Age-Related Shock Index (ASI) at admission (35.53 ± 17.82) compared with those who did not require transfusion (27.89 ± 9.53) (t(61) = -2.32, p = 0.024).n

**Table 9:** Comparison of ISS, SI, and MSI between ICU and non-ICU patients Continuous variables were expressed as mean ± standard deviation and compared between groups using the independent two-tailed Student’s t-test. A p-value < 0.001 is considered highly statistically significant [13,14].

Variable	ICU Admission	Mean ± SD	Total Mean ± SD	Test Statistic	P-value
Injury Severity Score (0–75)	No	21.32 ± 5.72	31.29 ± 11.77	t(61) = –5.95	p < 0.001 (highly significant)
	Yes	35.59 ± 11.09			
Shock Index (SI)	No	0.83 ± 0.15	1.21 ± 0.36	t(61) = –9.37	p < 0.001 (highly significant)
	Yes	1.38 ± 0.29			
Modified Shock Index (MSI)	No	1.09 ± 0.20	1.53 ± 0.46	t(61) = –7.53	p < 0.001 (highly significant)
	Yes	1.73 ± 0.41			
Age-Related Shock Index (ASI)	No	27.89 ± 9.53	35.53 ± 17.82	t(61) = –2.32	p = 0.024 (significant)
	Yes	38.83 ± 19.57			

Table 10 presents a comparison between the ICU and non-ICU groups. A significantly higher proportion of ICU-admitted patients fell into the most severe categories across all indices (p < 0.001). These findings underscore the strong association between elevated ISS (>25), SI (≥1.4), and MSI (>1.3) with ICU admission, reinforcing their relevance as early markers for critical care triage in trauma patients.

**Table 10:** Distribution of Injury Severity Score (ISS), Shock Index (SI), and Modified Shock Index (MSI) across ICU and non-ICU trauma patients Chi-square tests showed significant associations between ICU admission and ISS (χ² = 14.09, df = 2, p < 0.001), SI (χ² = 26.15, df = 2, p < 0.001), and MSI (χ² = 19.95, df = 1, p < 0.001) [11-14].

Variables	ICU		Total, N=63	Test Statistic	P-value
	No	Yes			
Injury Severity Score (75)					
1–8	0 (0%)	0 (0%)	0 (0%)	χ² = 14.09, df = 2	<0.001 (highly significant)
9–15	2 (10.5%)	1 (2.3%)	3 (4.8%)		
16–24	10 (52.6%)	6 (13.6%)	16 (25.4%)		
>25	7 (36.8%)	37 (84.1%)	44 (69.8%)		
Shock Index( SI)					
<0.6	0	0	0	χ² = 26.15, df = 2	<0.001 (highly significant)
0.6–1.0	15 (78.9%)	8 (18.2%)	23 (36.5%)		
1.0–1.4	4 (21.0%)	7 (15.9%)	11 (17.5%)		
≥1.4	0	29 (65.9%)	29 (46.0%)		
Modified Shock Index (MSI)					
<0.7	0	0	0	χ² = 19.95, df = 1	<0.001 (highly significant)
0.7–1.3	16 (84.2%)	9 (20.5%)	25 (39.7%)		
>1.3	3 (15.8%)	35 (79.5%)	38 (60.3%)		

The discriminative ability of MSI to predict blood transfusions was assessed using ROC curve analysis. The area under the receiver operating characteristic curve (AUROC) was 0.890 (SE 0.0421, p<0.001), indicating excellent diagnostic performance.

**Figure 1:**
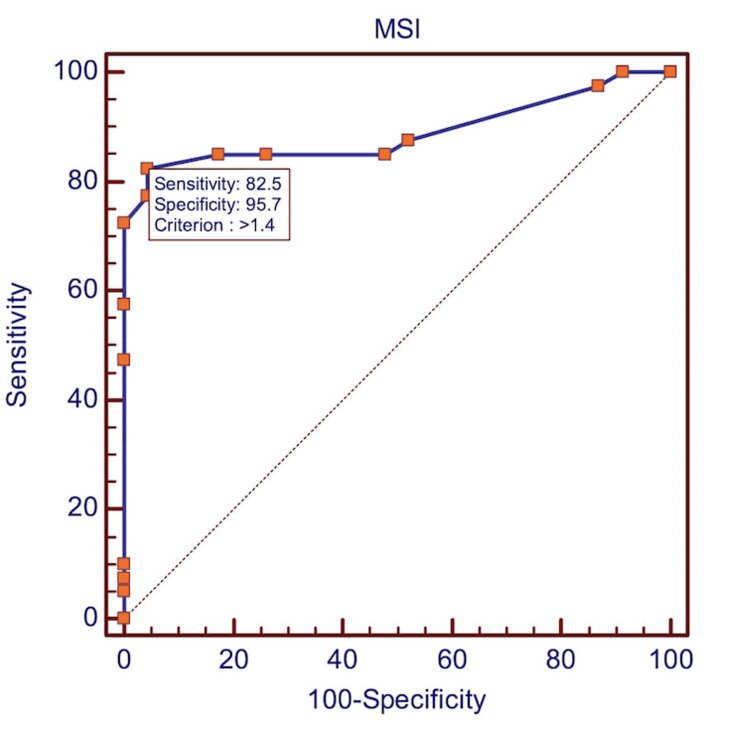
ROC curve analysis: MSI as a predictor for blood transfusion. ROC curve of modified shock index (MSI) for predicting blood transfusion requirement, showing AUROC 0.890; SE 0.0421; p<0.001, highly statistically significant [12-15].

The ROC analysis (Table 11, Figure 1) confirmed MSI as a highly accurate predictor of transfusion, with an AUROC of 0.890. An MSI cutoff >1.40 provided 82.5% sensitivity and 95.65% specificity, indicating strong discriminatory power. The positive likelihood ratio (LR+) of 18.98 means patients with MSI above this threshold have nearly 19 times higher odds of needing transfusion, while a negative LR of 0.18 supports MSI's utility in ruling out massive hemorrhage.

**Table 11:** Receiver operating characteristic (ROC) analysis for MSI predicting transfusion requirement Test statistic. AUROC (χ² test vs. null hypothesis AUROC = 0.5): 0.890 (SE = 0.0421, p < 0.001), indicating excellent discriminative performance. Data are presented as percentages unless otherwise specified [13-15]. SE: Standard Error; AUROC: Area Under ROC; LR+: Likelihood Ratio Pos; LR-: Likelihood Ratio Neg

Variables	ROC Results to Predict Blood Transfusions				Cut-off	AUROC (Test Statistic)	SE	P-value
	Sensitivity	Specificity	LR+	LR-				
MSI	82.5	95.65	18.98	0.18	>1.40	0.89	0.0421	<0.001

As shown in Table 12, both MSI >1.3 and blood loss ≥1500 mL were significant predictors of transfusion (p < 0.001). Although longer hospital stays (>5 days) occurred more frequently among transfused patients, this difference was not statistically significant (p = 0.571). These findings underscore MSI and hemorrhage volume as key early indicators for transfusion need and clinical severity.

**Table 12:** Correlation between transfusion requirement, Modified Shock Index (MSI), blood loss, and length of hospital stay (LOHS) Statistical tests: Chi-square test for LOHS (χ² = 3.85, df = 5, p = 0.572, NS) and blood loss (χ² = 36.00, df = 3, p < 0.001, highly significant); Fisher's Exact test for MSI (OR = ∞, p < 0.001, highly significant [8,10-12].

Variables	Blood Transfusion		Total	Test Statistic	P-value
	No	Yes			
LOHS					
0	1 (4.3%)	2 (5%)	3 (4.8%)	χ² = 3.85, df = 5	p = 0.571 (NS)
1–2	9 (39.1%)	8 (20%)	17 (27%)		
3–5	8 (34.8%)	13 (32.5%)	21 (33.3%)		
6–10	3 (13%)	11 (27.5%)	14 (22.2%)		
11–20	1 (4.3%)	3 (7.5%)	4 (6.3%)		
>20	1 (4.3%)	3 (7.5%)	4 (6.3%)		
Blood Loss (mL)					
0–750 mL	16 (69.6%)	3 (7.5%)	19 (30.2%)	χ² = 36.00, df = 3	p < 0.001 (highly significant)
750–1500 mL	7 (30.4%)	8 (20%)	15 (23.8%)		
1500–2000 mL	0 (0%)	15 (37.5%)	15 (23.8%)		
>2000 mL	0 (0%)	14 (35%)	14 (22.2%)		
Total	23 (100%)	40 (100%)	63 (100%)		
Modified Shock Index (MSI)					
<0.7	0 (0%)	0 (0%)	0 (0%)	Fisher’s Exact OR = ∞	p < 0.001 (highly significant)
0.7–1.3	19 (100.0%)	6 (13.6%)	25 (39.7%)		
>1.3	0 (0.0%)	38 (86.4%)	38 (60.3%)		

Table 13 presents the results of multivariable logistic regression. The MSI (MSI >1.3) was a significant independent predictor of massive transfusion, with an adjusted odds ratio of 11.42 (95% CI: 1.19-109.93, p = 0.035), indicating markedly higher odds of blood loss >1500 mL compared with MSI ≤1.3. Neither the ISS (OR: 0.98, 95% CI: 0.92-1.05, p = 0.645) nor age (OR: 0.94, 95% CI: 0.87-1.01, p = 0.107) was a statistically significant predictor.

**Table 13:** Multivariate logistic regression coefficients (β, log‑odds scale) for blood loss >1500 mL from MSI>1.3, ISS, and age MSI > 1.3: |z| = 2.10, p = 0.035 (significant), CI = 1.19 – 109.93 [10-15].

Multivariate Logistic Regression Results With Wald Test (Dependent: Blood loss >1500 mL)						
Predictor	OR (=e^β)	95% CI (Low–High)	β (Coef)	SE(β)	z-statistic	p-value
MSI >1.3	11.42	1.19–109.93	2.435	1.159	2.1	0.035
ISS	0.98	0.92–1.05	-0.015	0.033	-0.46	0.645
Age	0.94	0.87–1.01	-0.061	0.037	-1.61	0.107
Constant	0.42	0.02–9.67	-0.872	1.602	-0.54	0.587

**Figure 2:**
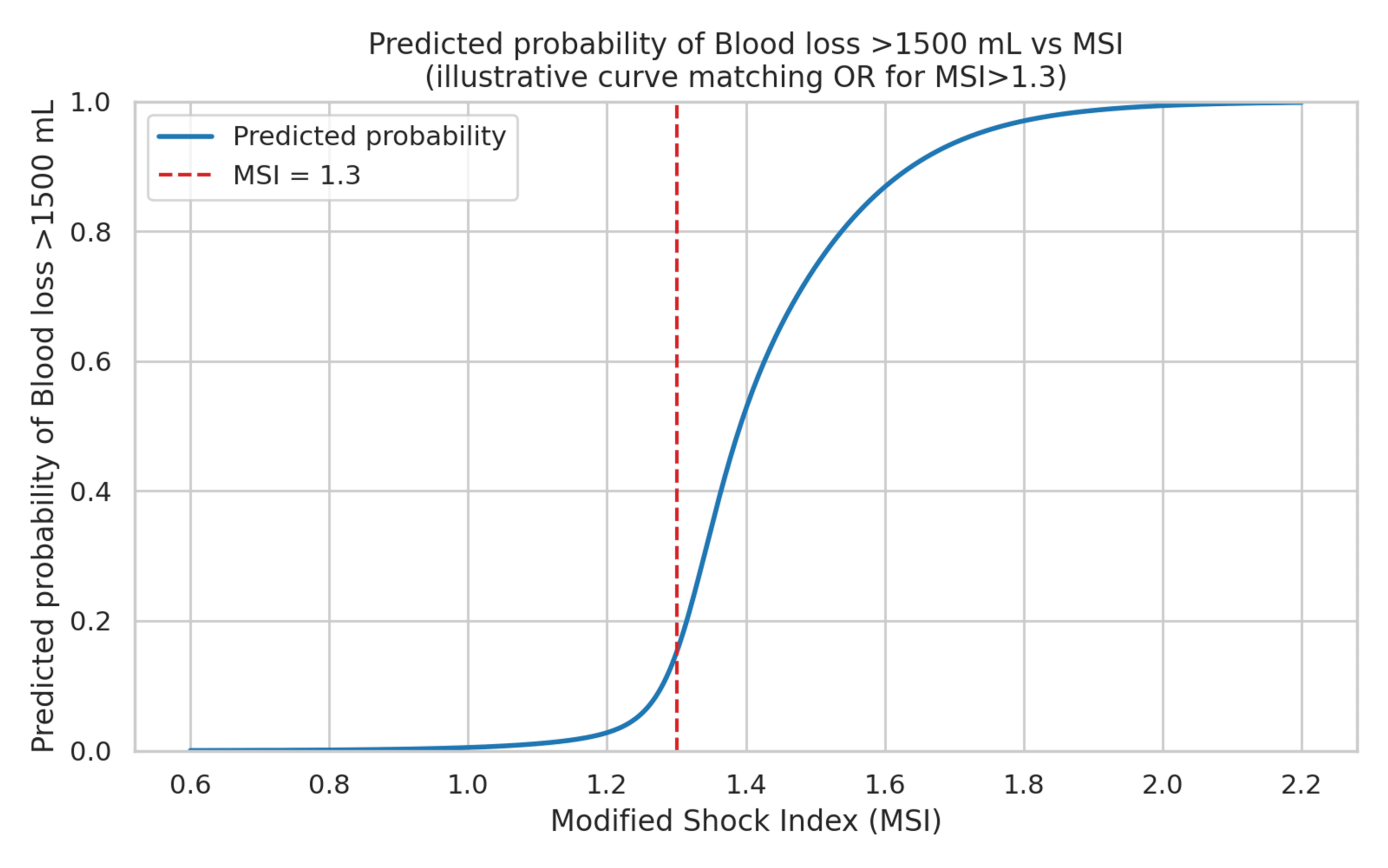
Logistic S-curve for MSI analysis. Dependent variable (Outcome) = Blood loss >1500 mL (binary: Yes/No) • Independent variables (Predictors) = • MSI >1.3 (key predictor, significant) • ISS (covariate) • Age (covariate) • X-axis = Modified Shock Index (MSI) • Y-axis = Predicted probability of Blood loss >1500 mL • Adj OR (MSI > 1.3) = 11.42 • 95% CI = 1.19 – 109.93 • p = 0.035 predicted probability curve will look like an S-shaped logistic curve, very flat at low MSI, then rising steeply around MSI = 1.3, and approaching high probability values above [10-15].

Figure 2 shows the logistic S-curve for MSI analysis. An adjusted odds ratio of 11.42 for MSI >1.3 indicates that patients above this threshold were approximately 11 times more likely to experience blood loss >1500 mL and, therefore, at a substantially higher risk of requiring a blood transfusion compared with those at or below 1.3, after adjusting for ISS and age. Although the wide 95% CI (1.19-109.93) reflects considerable uncertainty in the magnitude of effect, likely due to small sample size, the p-value (0.035) confirms statistical significance and highlights the bedside utility of MSI >1.3 as a rapid, non-invasive marker to identify trauma patients who may need early and aggressive transfusion [10-15].

## Discussion

Initial research established the SI as a dynamic predictor of mortality and massive transfusion, particularly among older patients and those with blunt trauma, although its correlation with outcomes varied across clinical settings [16]. Guly et al. critically evaluated trauma registry data and demonstrated that traditional ATLS shock classifications may overstate the relationship between vital signs and blood loss. While tachycardia is associated with increased mortality, its absence does not exclude shock, as significant hemorrhage can present with bradycardia. A comprehensive assessment should therefore incorporate physiological parameters, injury patterns, and biochemical markers such as lactate, with further research needed to develop evidence-based methods for estimating blood loss and guiding resuscitation [17].

Hasler et al. identified systolic blood pressure ≤110 mmHg as significantly associated with increased mortality in penetrating trauma, highlighting the limitations of conventional thresholds and the necessity for more sensitive physiological markers [18]. Mutschler et al. validated the predictive value of SI for transfusion needs while recommending its incorporation into multi-parameter frameworks, thereby supporting the MSI as a more refined and clinically practical tool [19]. Rao and Martin emphasized the importance of structured, evidence-based protocols in massive hemorrhage management, reinforcing the utility of early indices such as SI and MSI for guiding timely transfusion decisions [20]. Collectively, these findings reflect a paradigm shift from reliance on static vital sign thresholds to the use of composite indices such as the MSI, which improves early recognition of high-risk trauma patients, enhances triage accuracy, and guides timely resuscitative interventions.

The current study further affirms MSI as a clinically valuable, noninvasive tool for predicting transfusion needs in trauma care. An MSI >1.3 was significantly associated with higher rates of transfusion (63.5%), ICU admission (69.8%), and mechanical ventilation (49.2%). No patients had MSI < 0.7, indicating strong discriminative ability. These results are consistent with previous studies that demonstrated MSI's superior prognostic accuracy over the traditional SI, particularly in predicting mortality and ICU admission [21]. While the majority of patients in our cohort were young adults (47.6%), other studies have highlighted MSI's predictive performance in elderly trauma populations [22-24].

In this cohort, an MSI threshold of >1.40 demonstrated excellent predictive accuracy for transfusion, with an AUROC of 0.890 (95% CI: 0.807-0.973), sensitivity of 82.5%, and specificity of 95.65%, consistent with international findings [25-28]. Diagnostic strength was underscored by robust likelihood ratios (LR+: 18.98, LR−: 0.18), indicating that patients with MSI > 1.40 had nearly 19-fold higher odds of requiring transfusion, while values ≤ 1.40 strongly argued against it. Predictive values were similarly high (PPV 94.2%, NPV 86.5%), reinforcing its clinical utility in guiding early transfusion decisions. Collectively, these findings establish MSI >1.40 as an excellent threshold for early risk stratification and transfusion planning, underscoring its potential role in trauma protocols to facilitate timely resuscitation and reduce complications, such as trauma-induced coagulopathy. Early identification of transfusion needs is therefore critical in mitigating adverse outcomes, echoing the observations of prior studies [29]. Taken together, our results support the integration of dynamic hemodynamic indices, such as MSI, into trauma care protocols to enhance rapid and accurate clinical decision-making.

Study limitations

This study has several limitations. The relatively small sample size restricts statistical power and generalizability, emphasizing the need for larger, multicenter studies to validate these findings. The cohort was predominantly young adult males (68.2% aged 20-40 years; 85.7% male), introducing demographic bias and limiting applicability to older adults and females. Potential documentation errors and uncontrolled prehospital factors, such as fluid resuscitation and transport time, may have contributed to inherent biases.

Key confounders, including mechanisms of injury and pre-existing comorbidities, were not considered and may have influenced initial vital signs and transfusion decisions. Excluding patients who died within six hours could have introduced survivorship bias, potentially overestimating specificity and AUROC and limiting applicability to the most critically unstable trauma patients. The MSI cutoff used (>1.3) may not generalize across diverse clinical settings, and wide confidence intervals in regression analyses reflect limitations related to sample size and clinical heterogeneity. The focus on in-hospital outcomes excludes long-term complications, and outcomes such as transfusion or ICU admission may have been influenced by the indices under evaluation, introducing potential circular reasoning.

While MSI demonstrated predictive value, it should not be considered an independent predictor of morbidity or mortality but rather a supportive adjunct to comprehensive clinical assessment. Future studies should validate MSI thresholds across diverse populations, account for confounders, compare with other indices, assess long-term outcomes, minimize bias, and explore integrating MSI into trauma care protocols.

## Conclusions

The MSI has demonstrated strong predictive value in identifying patients requiring transfusion, particularly massive transfusion, in trauma and hemorrhagic scenarios. Our study indicates that elevated MSI is associated with increased transfusion requirements, a higher mortality risk, and a greater likelihood of critical care admission. While MSI provides a practical bedside tool with sensitivity and specificity comparable to the traditional SI, its clinical utility has yet to be fully standardized. Further research is needed to validate optimal MSI thresholds and to integrate the index effectively into transfusion decision-making protocols.
